# A new single-step protocol for rapid baculovirus-driven protein production in insect cells

**DOI:** 10.1186/s12896-017-0400-3

**Published:** 2017-11-16

**Authors:** Judith Scholz, Sabine Suppmann

**Affiliations:** Max-Planck Institute of Biochemistry, Am Klopferspitz 18, 82152 Martinsried, Germany

**Keywords:** Baculo expression vector system BEV, Insect cells, Protein expression, Transfection, Virus amplification

## Abstract

**Background:**

In the last three decades, the Baculovirus expression vector system (BEV) has evolved to one of the most widely used eukaryotic systems for heterologous protein expression including approved vaccines and therapies. Despite the significant improvements introduced during the past years, the BEV system still has major drawbacks, primarily the time required to generate recombinant virus and virus instability for certain target proteins. In this study we show that the conventional method to generate recombinant Baculovirus using a Tn7 transposition based system can be shortened to a single-step transfection-only procedure without further amplification.

**Methods:**

In a first step we have adapted a recently published protocol that replaces the standard liposome-based transfection procedure of adherent insect cells by transfecting insect cells in suspension with a preformed DNA-PEI complex generating P0 virus. We have then expressed and purified six different target proteins, among them four intracellular and two secreted proteins, by infecting insect cells either with P0 or P1 virus.

**Results:**

We demonstrate that transfection in suspension is as efficient as the standard protocol, but in addition allows generation of high amounts of P0 virus early in the process. To test if this P0 virus generated by bacmid transfection can be used directly for protein expression in either the screening or production process, we compared P0 versus amplified P1 virus-mediated protein expression. We show that protein expression levels, purity and yield of the purified proteins are equally high for P0 and P1.

**Conclusion:**

The standard protocol for generating recombinant baculovirus comprises transfection of the bacmid followed by one or two subsequent virus amplification steps. In this study we show that Baculovirus generated by transfection-only is equally efficient in driving protein expression. This reduces the time from bacmid DNA to protein to eight days and reduces the risk of virus decay. In contrast to transient gene expression protocols, the required amount of DNA is minimal: 100 µg bacmid DNA is sufficient for a production scale of 10 L.

## Background

**Fig. 1:**
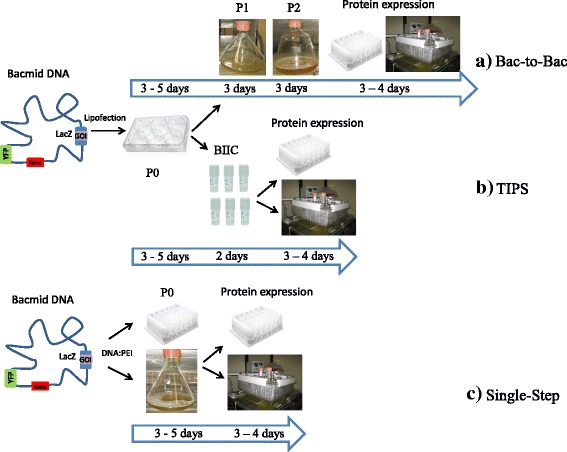
Comparison of different baculovirus expression strategies using Tn7 transposon mediated gene integration. The recombinant EMBacY bacmid carries the integrated gene of interest GOI and YFP for visualization. **a** Conventional Bac-to-Bac procedure with transfection of adherent cells to generate P0 baculovirus followed by one or more subsequent virus amplification steps P1and P2. **b** Titerless infected-cells preservation TIPS protocol. Insect cells infected with P0 virus are frozen prior to cell lysis and used for protein expression in screening and production. **c** Single-step procedure presented in this study. P0 virus is directly used for expression screening and production

Since its first publication more than 30 years ago [1], recombinant baculoviruses today are used for a broad range of applications, predominantly for heterologous protein expression in basic and applied research [2–4], for virus-like particles commercialized as vaccines [5–8] and as gene delivery vectors for mammalian cells [9], [reviewed in 2]. The power of the baculoexpression vector system for heterologous protein expression has two reasons. First, two strong promotors polyhedrin polH and p10 of the baculovirus *Autographa californica* multicapsid multinucleohedrovirus (AcMNPV) drive target protein expression to high levels, while the respective baculogenes are dispensible for virus propagation. Second, the lepidopteran cell lines from *Spodoptera frugiperda* (Sf21 and S9) and *Trichoplusia ni* (Hi5) that are mainly used for infection, can grow to high cell densities in suspension and are capable of introducing post-translational modifications including N- and O-linked glycosylation as well as assembling multiprotein complexes [10, 11]. Two different strategies for target gene integration into the Baculovirus genome have been developed and commercialized. The Bac-to-Bac principle (DH10Bac Invitrogen, Multibac and EMBacY [4]) uses Tn7 transposition of the target gene from the corresponding transfer vector into the baculovirus genome within *E.coli* cells already carrying the virus genome. Recombinant baculovirus is selected by blue-white screening and the bacmid DNA can further be analyzed for correct gene integration excluding the risk to propagate non-recombinant virus. The BagMagic™ (Novagen) and *flash*BAC™ (Oxford Expression Technologies) systems generate recombinant baculovirus directly in insect cells by cotransfecting transfer vector and bacmid DNA. The non-recombinant viral DNA is replication-incompetent, allowing propagation of recombinant virus only. Although recombinant virus generation is less time consuming with these systems, the fact that gene integration cannot be monitored and the high costs of the systems prompted us to use a Tn7 transposition-based virus. The major disadvantage here is that baculovirus generation can take two to three weeks, depending on the number of virus amplification steps (Fig. 1). Recently published approaches like transient gene expression TGE [12, 13] and TGE in combination with coinfection/transactivation [14, 15] have sped up the process considerably. While these strategies address predominantly construct screening, the titerless infected-cells preservation and scale-up (TIPS) method streamlines the entire process from screening to large scale production [16]. Baculovirus-infected insect cells (BIIC) are harvested and frozen prior to cell lysis, when virus would be released into the culture supernatant. These cell stocks, densely packed with recombinant baculovirus, are used to infect insect cells for small-scale screens as well as large-scale productions with as little as 1 mL of BIIC needed for a 100 L scale ([17], Fig. 1). The TIPS method eliminates repeated virus amplification steps and at the same time provides a long-term stable storage form of recombinant baculovirus, which is particularly important for unstable virus stocks. For these reasons, we have implemented this methodology several years ago. As the central Biochemistry Core Facility at the Max-Planck Institute of Biochemistry, we provide in-house services for recombinant protein production for different departments and research groups. We use the baculovirus expression system for diverse and difficult-to-express targets, with one new target every 2 months in the pipeline. In contrast to the reported five-year stability of BIICs we have noticed instability of cell stocks for certain target proteins, eg Vasp [18] and Cab45 [19], with considerable drop in expression levels 3 months after freezing. In search for alternative protocols that shorten virus generation and circumvent any stability issue, the recently published protocol for transfecting insect cells with bacmid DNA in suspension with a preformed DNA:PEI complex [20] opened a new route: the optimization, standardization and upscale of baculovirus generation in a cost-efficient and high throughput-compatible procedure. However, this P0 virus is generally assumed to have low titers and to need further virus amplification steps to generate P1, P2 etc. The idea presented in this study to use P0 directly in screening and production is based on our observation, that P0 virus titers are on the contrary rather high. Using the Sf9 Easy Titer cell line [21] we have measured P0 titers for different proteins in the range of 5.2 × 10^7^ PFU / ml to 1.9 × 10^9^ PFU / ml with an average titer between 2.9 and 4.9 × 10^8^ PFU / ml. The purpose of this study was to compare protein expression in insect cells infected either with P0 or P1. For this validation we have expressed and purified six different target proteins, among them four intracellular proteins in the range of 68 kDa to 204 kDa, including difficult-to-express and degradation-prone proteins and two secreted proteins 19 kDa and 76 kDa in size.

## Results

### Generation of recombinant P0 and P1 baculovirus

**Fig. 2:**
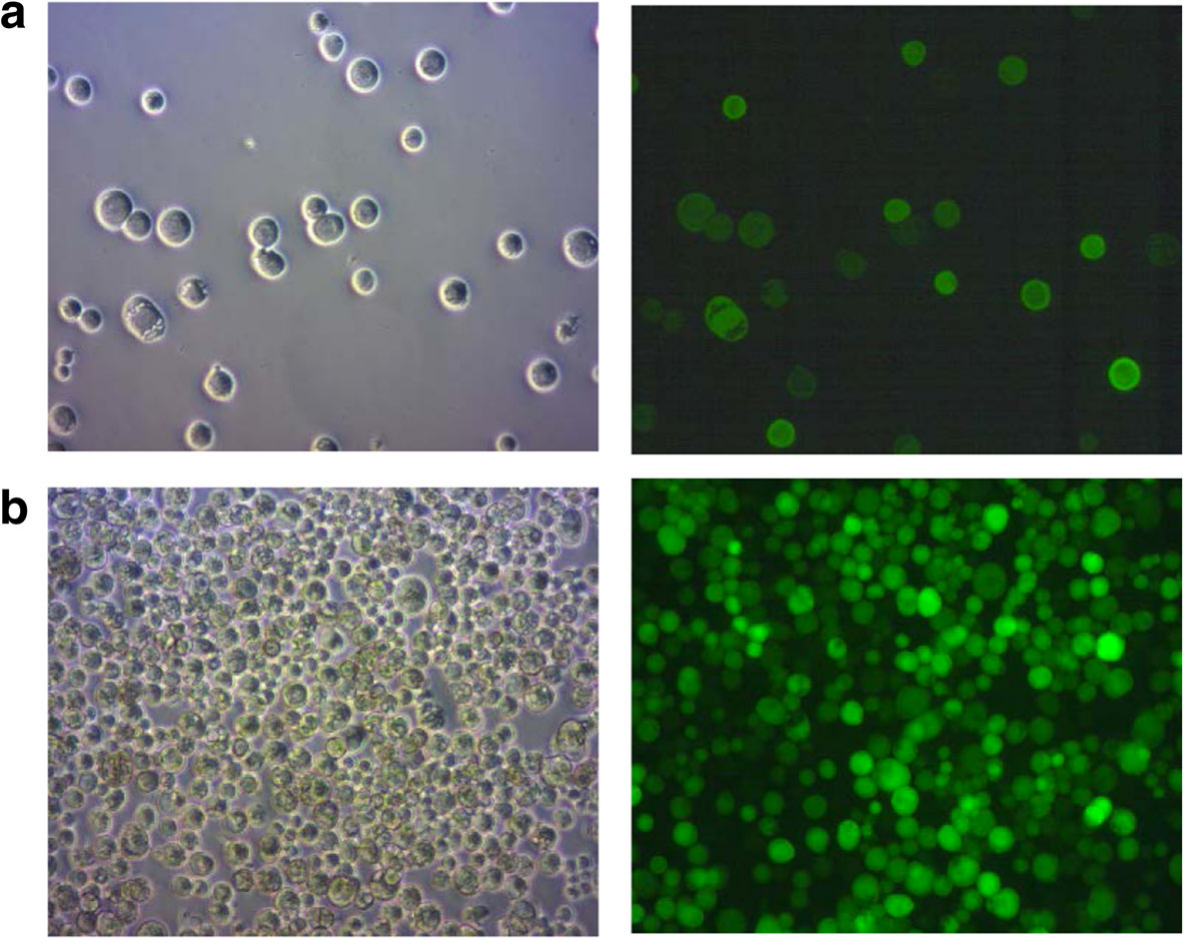
Transfection in suspension versus transfection of adherent cells. Brightfield and YFP fluorescence of (**a**) Sf9 suspension cells transfected with EMBacY construct #1, d5 post transfection, DNA:PEI = 1:2; (**b**) Sf9 adherent cells transfected with with EMBacY construct #1, d4 post transfection; transfection was performed with Cellfectin

The experiments described in this study were performed with EMBacY baculovirus (Fig. 1, [4]), a derivative of the Multibac bacmid with an integrated enhanced yellow fluorescence protein-coding gene (YFP) that allows to monitor virus production with high sensitivity using either fluorescence microscopy or spectrophotometry. Moreover, YFP is under control of a very late polyhedrin polH promotor and thus serves as a surrogate marker for expression of the heterologous protein which is also under control of a late promotor p10. The parental Multibac virus, originally tailored for multigene assembly is an attractive choice also for single proteins due to the deletion of *v-cath*, a viral protease and its activator *chiA*, that substantially reduces proteolysis of overexpressed proteins [11]. Figure 2 shows the comparison of adherent Sf9 cells transfected with Cellfectin versus Sf9 suspension cells transfected with a preformed DNA:PEI complex of 1 μg EMBacY bacmid DNA and 2 μl PEI / ml culture. This qualitative microscopy analysis shows that transfection in suspension is at least as efficient as the conventional method. The fact that suspension cells are amenable to analysis of viability and diameter opens the additional option to optimize and standardize the transfection step with these parameters. First of all, we have correlated cell size, viability and YFP fluorescence with protein productivity in a series of test expressions (data not shown). P0 baculovirus was shown to be most productive when Sf9 cells at transfection harvest have a viable cell count of 0.6–0.9 × 10^6^ cells /ml, viability of 65% - 90%, and diameter of 25–26 μm compared to 0.8, > 91% and 21 μm for control cells, cell count being the most relevant parameter. Next, based on these parameters, the optimal ratio of DNA:PEI was tested using a fixed amount of bacmid DNA (1 μg bacmid DNA/ml culture) shown to be optimal in previous experiments. Table 1 shows cell count, viability and diameter of Sf9 cells transfected with DNA:PEI complexes at ratios of 1:2, 1:4 and 1:6 exemplified for construct #1 and #2. The ratio of DNA:PEI = 1:2 best fulfilled the optimal cell parameters and was chosen to generate P0 baculovirus for constructs #1 to #6. Next, the harvest time point of transfection had to be optimized. According to the authors of EMBacY the initial virus (V_0_ corresponding to P0 used in this manuscript) is harvested no later than 48–60 h post-transfection and then amplified at low multiplicity of infection to prevent detrimental gene deletions [4]. However, we typically hardly detect significant signs of infection like increased cell size or drop in viability at 48–60 h post-transfection, probably due to low transfection efficiencies. We therefore routinely harvest transfections of insect cells when signs of infection are visible, which is in the range of 3 to 5 days post-transfection depending on the target protein. For the proteins expressed in this study, 5 days post-transfection was shown to be the most appropriate harvest time for all constructs. This prolonged transfection time implies, that the initial virus generated by transfection may have undergone amplification. Strictly speaking, the term “P0” used throughout this study describes the product of a prolonged transfection that may include amplified virus. In order to optimize productivity of P1 baculovirus, the amount of P0 virus used for the amplification step had to be adjusted to induce cell parameters as described before. Again a series of experiments (data not shown) revealed that the optimal amount of P0 can be derived from YFP fluorescence of transfected cells. As a rule of thumb, a dilution of 1:1000 (1 μl P0 / ml Sf9 cells) at low transfection efficiency of 50% to 1:5000 at high efficiency >90% is appropriate to generate productive P1 baculovirus. Table 1 shows cell count, viability and diameter of Sf9 cells infected with P0 at 2500- and 5000-fold dilution exemplified for construct #1 and #2. Accordingly, a 3000-fold dilution of P0 was chosen to amplify recombinant baculovirus for constructs #1 to #6. Table 2 summarizes parameters of Sf9 cells at harvest of either P0 and P1 d5 post-transfection and d4 post-infection respectively for all constructs used in this study. The data are almost comparable for the different target proteins within the P0 and P1 series except construct #6, for which viability or cell count dropped below the optimal level described before. As this target protein was an outlier in both virus series we nevertheless used this P0 and P1 for the following experiments.

**Table 1:** Optimization of P0 and P1 virus generation

Construct	DNA:PEI	Cell count × 10^6^	Viability at harvest (%)	Diameter at harvest (μm)
Cells before transfection*		0.8	91.5	21.2
#1	1:2	1.0	91.1	23.1
#1	1:4	0.7	75.2	22.1
#1	1:6	0.5	59.4	21.4
#2	1:2	1.0	89.0	25.3
#2	1:4	0.7	81.0	24.2
#2	1:6	0.4	70.0	22.0
Construct	P0 dilution	Cell count × 10^6^	Viability at harvest (%)	Diameter at harvest (μm)
#1	2500	0.6	74	23.7
#1	5000	1.0	78.4	24.6
#2	2500	0.4	62.4	24.1
#2	5000	0.9	74.1	24.9

Sf9 cell parameters for different DNA:PEI ratio and different P0 dilutions used for P1 generation; * Sf9 cells were split 2–3 h prior to transfection or infection; data were recorded immediately before transfection or infection

**Table 2:** P0 and P1 virus generation at harvest

Construct	Virus	Cell count × 10^6^	Viability (%)	Diameter (μm)
#1	P0	0.8	63.7	25.0
#2	P0	0.9	66.5	26.2
#3	P0	0.7	56.5	24.9
#4	P0	0.9	68.4	24.4
#5	P0	1.2	72.6	25.9
#6	P0	0.7	48.2	23.3
#1	P1	0.6	72.6	28.5
#2	P1	0.7	75.9	28.6
#3	P1	0.6	74.3	28.8
#4	P1	0.8	81.4	28.7
#5	P1	0.7	79.5	28.6
#6	P1	0.5	63.8	28.1

### Small scale optimization of protein expression

**Fig. 3:**
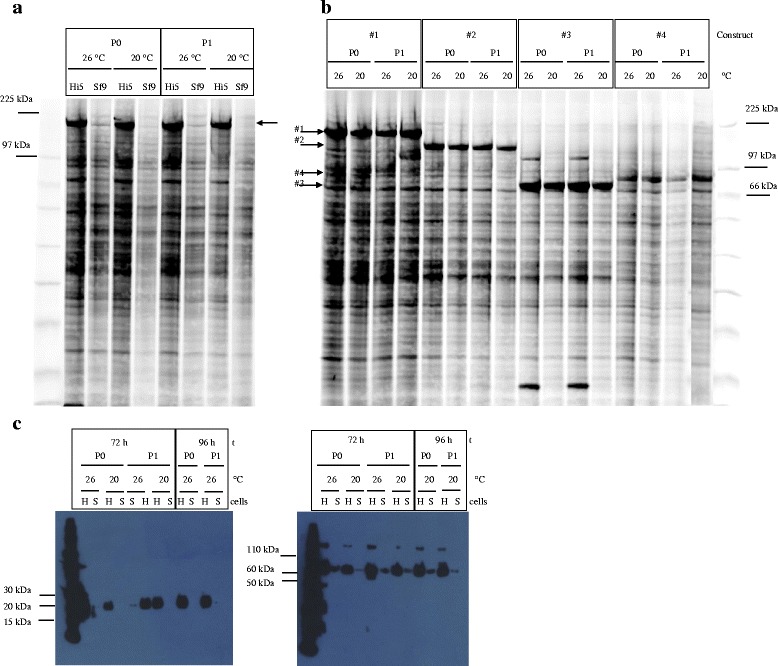
**a** P0 versus P1 total cell lysates of construct #1 expressed in Sf9 and Hi5 cells at 20 °C and 26 °C for 72 h visualized by Cy5 labelling of Easy SDS-PAGE (**b**) P0 versus P1 total cell lysates of construct #1, #2, #3, #4 expressed in Hi5 cells at 20 °C and 26 °C for 96 h visualized by Cy5 labelling and Easy SDS-PAGE; expression levels of total cellular protein [%] for (A) and (B) are depicted in Table 3; (**c**) P0 versus P1 culture supernatant of construct #5 and #6 expressed in Sf9 (S) and Hi5 (H) cells at 20 °C and 26 °C for 72 h or 96 h, loaded on 4–12% Bis-Tris Nu-PAGE and visualized by Western Blotting using HisProbe™-HRP

The parameters used to optimize protein expression were a combination of Sf9 or Hi5 cells; 20 °C or 26 °C expression temperature and 72 h or 96 h expression time. The amount of P0 or P1 virus used for infection was not varied. Based on previous experiments, 80–100 μl P0 or P1 virus / 10 ml expression culture resulted in maximal YFP fluorescence as well as target protein expression and was used for all expression experiments shown in this study. Total lysates of construct #1, #2, #3 and #4 were analyzed with the Amersham Easy SDS-PAGE system using Cy5 staining of the samples and subsequent quantification. This allows the quantitative comparison of expression levels with a threshold of ca 2.5%. As Cy5 staining is based on lysine labelling, such a quantitation is only meaningful for the same protein under different conditions, as performed in this study. Fig. 3a, b and Table 3 summarize the results for the four intracellular proteins. For all constructs and conditions tested, P0 and P1 mediated protein expression was equally high. The cell line used for infection had substantial impact on expression levels. Depending on the construct, Hi5 cells expressed 2–10 fold higher levels compared to Sf9, as shown in Table 3 and exemplified for construct #1 in Fig. 3a. Results for construct #1 and #2 in Hi5 cells are especially striking, as these two proteins can be considered as difficult-to-express: more than 50% of the participants of a recent benchmarking study failed to achieve detectable expression (manuscript in preparation). Expression driven by P0 virus of construct #1 and #2 reached up to 47% and 44% of total cellular protein, expression driven by P1 virus 54% and 37% respectively (Fig. 3b, Table 3). The other two constructs #3 and #4 were also expressed at considerable levels: expression driven by P0 virus reached up to 59% and 28% of total cellular protein, expression driven by P1 virus 61% and 27%, respectively (Fig. 3b, Table 3). With regard to temperature and expression time, 20 °C and 96 h yielded higher expression levels for almost all intracellular constructs (Fig. 3a, b and Table 3). In contrast to the intracellular targets, protein levels of the two secreted proteins #5 and #6 in culture supernatants were not high enough for significant detection in Cy5 SDS-PAGE. These samples were instead analyzed by Western Blotting (Fig. 3c). As for the intracellular proteins, expression levels were comparable for both P0 and P1 virus and much higher in Hi5 than Sf9 cells. However, temperature and expression time had almost no impact on expression level. Based on the expression results, conditions chosen for the production were Hi5 cells, 20 °C and 96 h expression time.

**Table 3:** Expression levels of P0 and P1mediated expression

Construct	cells	Temp [°C]	% of TCP* P0	% of TCP* P1
for	72 h			
#1	Hi5	26C	31.6	45.9
#1	Hi5	20C	37.9	53.7
#1	Sf9	26C	3.5	2.5
#1	Sf9	20C	nd	6.1
#2	Hi5	26C	43.7	32.3
#2	Hi5	20C	34.5	34.4
#2	Sf9	26C	10.5	nd
#2	Sf9	20C	26.6	nd
#3	Hi5	26C	52.4	51.9
#3	Hi5	20C	58.7	38.4
#3	Sf9	26C	19.1	3.3
#3	Sf9	20C	12.5	7.2
#4	Hi5	26C	17.5	26
#4	Hi5	20C	23.6	26.2
#4	Sf9	26C	5.9	4
#4	Sf9	20C	8.5	7.7
for	96 h			
#1	Hi5	26C	44.9	31
#1	Hi5	20C	46.6	46.7
#2	Hi5	26C	31.5	27.9
#2	Hi5	20C	40.1	36.9
#3	Hi5	26C	54.1	47.7
#3	Hi5	20C	56.7	61.1
#4	Hi5	26C	19	24.5
#4	Hi5	20C	28.1	26.8

Expression levels [%] of *total cellular protein derived from Cy5 quantification of P0 versus P1 total cell lysates of construct #1, #2, #3, #4 expressed in Sf9 and Hi5 at 20 °C and 26 °C for 72 h and in Hi5 for 96 h

### Protein production

**Fig. 4:**
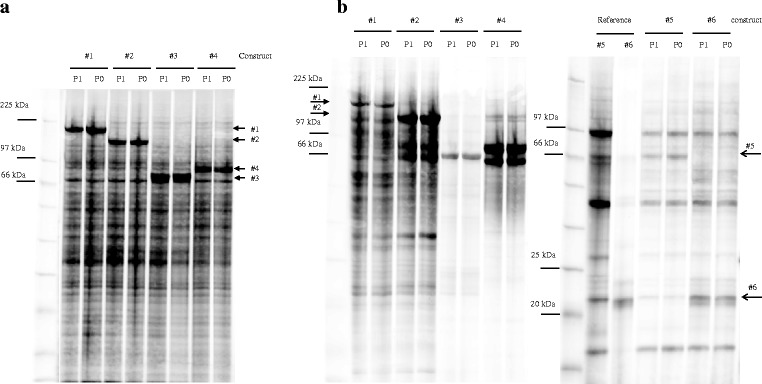
**a** P0 versus P1 total cell lysates of 100 ml scale productions of construct #1, #2, #3, #4 in Hi5 cells at 20 °C for 96 h visualized by Cy5 labelling loaded on Easy SDS-PAGE. **b** construct #1 to #6 purified by one-step IMAC chromatography visualized by Cy5 labelling loaded on Easy SDS-PAGE; reference samples of #5 and #6 from previous one-step IMAC were loaded as control, migration position is indicated

**Fig. 5:**
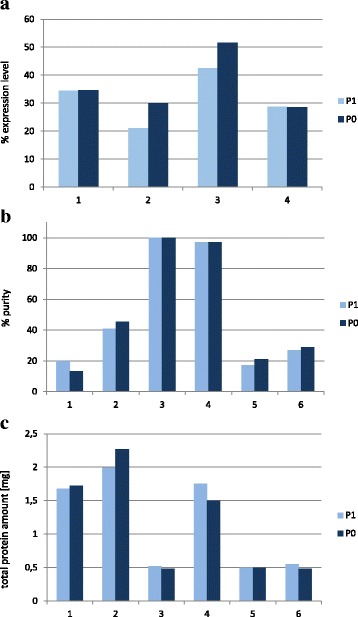
**a** Expression levels [%] of total cellular protein derived from Cy5 quantification of Fig. 3a; (**b**) % protein purity derived from Cy5 quantification of Fig. 3b; (**c**) total protein yield from protein production at 100 ml scale for constructs #1- #4 and 200 ml scale for constructs #5 und #6 as determined by Bradford staining

Although the analysis of expression levels in total lysates were very promising, they are of limited informative value, because they don’t include any information on protein solubility, stability etc. To address these questions, we have purified constructs #1 to #6 from 100 to 200 ml productions performed according to the optimization results. Expression levels of #1 to #4 (Fig. 4a, Fig.5a) were again very high, but lower than in test expressions probably due to upscaling or virus decay. However, in agreement with test expression results, protein expression driven by P0 infected Hi5 cells was almost identical to P1 infected cells. All proteins were subsequently purified by one-step IMAC as described in Materials and Methods, and eluates were immediately loaded on Easy SDS-PAGE to quantify protein purity. Protein yield was determined by Bradford staining. Results are displayed in Fig. 4b 4c, Fig.5b and 5c. Consistent with low expression levels analysed by Western Blotting, yields and purity of the secreted proteins #5 and #6 were low with high amount of protein contaminants. Characterized reference samples of previous one-step purifications had to be loaded to confirm identity of the respective bands. Among the intracellular proteins, eluates of target #1 and #2, known to be difficult-to-express and degradation prone, showed only 20% purity containing a lot of degradation products. On the other hand construct #3 and #4 were purified almost to homogeneity in this single IMAC step. The most important result however is the fact, that six proteins, intracellular or secreted, different in size, solubility and stability, were purified at almost identical purity and yield from Hi5 cells either infected with P0 or P1 virus. This proves that P0 virus generated by bacmid transfection can be used directly for protein expression in either the screening or production process.

## Discussion

It has generally been assumed that baculovirus generated by transfection of insect cells with bacmid DNA (P0) has low titers and needs further amplification steps to produce titers high enough for protein expression. In this study we demonstrate the opposite: P0 virus titers are presumably high enough to drive protein expression up to 58% of total cellular protein and induced expression levels comparable to P1 virus for six different target proteins. Purification of all six proteins produced with either P0 or P1 by one-step affinity chromatography yielded equal amounts and purity, supporting the finding that a single transfection step to generate baculovirus is sufficient for protein production in insect cells. High amounts of P0 virus can be produced in a cost-efficient way by transfection of suspension cells with DNA:PEI complexes, as recently published [19]. PEI stocks are easy to produce in-house and can be stored at −20 °C. Low amounts of bacmid DNA are needed for protein expression, in contrast to transient transfection protocols. The amount of 100 μg bacmid DNA is sufficient to produce 100 ml recombinant baculovirus, which is sufficient to produce protein at a 10 L scale. The time from bacmid DNA to protein in either test expression or production is reduced to 8 days, which is shorter than any protocol described so far (Fig. 1). Even more important, along with the time saving, the problem of virus instability or decay of baculovirus-infected insect cells BIIC is eliminated with this new protocol, as the procedure starts with bacmid DNA. We have implemented this method for in-house projects up to 6 L scale successfully and - apart from BIICs as virus storage form for most target proteins - use exclusively bacmid DNA stored at 4 °C for target proteins with instable baculovirus or BIIC such as Cab45 or Vasp. In summary our new single-step procedure represents a valuable addition to the methodological improvements that have made baculovirus-mediated protein expression in insect cells one of the most important protein production platforms.

## Methods

### Constructs

For validation experiments, all target proteins were expressed as His fusion proteins cloned in pFastBac. The following gene numbering instead of names will be used throughout the text: #1 Dicer2 (mouse), 203.8 kDa, intracellular; #2 cAbl (human) 126 kDa, intracellular; #3 FMRP (human), 68 kDa, intracellular; #4 NS1-H1 (Parvovirus), 76 kDa, intracellular; #5 Soluble G protein (Hendra Virus), 76 kDa, secreted (gp67 signal sequence); #6 CSF1 (mouse), 19 kDa, secreted (honeybee melittin signal sequence). Construct #5 is C-terminally fused to His; all other constructs are N-terminally tagged.

### Cell culture

Sf9 (ATCC) or Hi5 cells (Gene Center, LMU Munich) were grown in suspension in serum-free Ex-Cell 420 (Sigma, Cat No 24420C) at 26 °C in shaker incubators (Infors, 50 mm rotating diameter) in Erlenmeyer EM glass flasks covered with Silicon Caps (Hirschmann, VWR) at the following combinations of culture volume - flask volume - shaking speed: 30 ml - 250 ml EM – 120 rpm; 60 ml – 0,5 L EM - 150 ml – 1 L EM – 120 rpm; 200 to 400 ml - 1.8 L FB – 90 rpm; 400 ml to 1 L – 5 L EM – 90 rpm. Cell count, viability and cell diameter were monitored on a Vi-CELL® instrument (Beckman Coulter, Germany). Cells were maintained at a density of 1–5 × 10^6^ cells/ml and 0.2–5 × 10^6^ cells/ml for Sf9 and Hi5 cells respectively. Cell diameter of uninfected cell lines is about 19 μm, and viability should be higher than 95%. It is important to ensure that cells are approximately those values in order to maintain high quality and reproducibility of experiments.

### Baculovirus expression system (BEVS)

Recombinant baculovirus was generated using the Tn7 transposition based EMBacY virus [4]. Correct gene integration was monitored by blue-white selection and confirmed by PCR using recombinant bacmid DNA as template using gene specific primers. Recombinant bacmid DNA was prepared using Nucleobond BAC100 (Macherey Nagel, Düren, Germany). The average yield from 100 ml *E.coli* culture was 100 μg bacmid DNA. For transfection of adherent cells, 2 ml Sf9 cells with 0.8 × 10^6^ cells in Ex-Cell 420 supplemented with 5% FCS were seeded per well of a 6-well plate 1–5 h prior to transfection. 1 μg bacmid DNA / ml culture was diluted in 100 μl serum-free ExCell 420 and vortexed gently. A mix of 8 μl Cellfectin (Invitrogen, Cat No 10362–010) in 100 μl serum-free ExCell 420 was slowly added to the DNA solution. Complex formation was allowed for 15–45 min at room temperature and the solution was then added dropwise to the cells. Cells were incubated at 26 °C for 3 to 5 h. Medium was then replaced by 2 ml Ex-Cell 420 supplemented with 5% FCS and incubated at 26 °C for 5–9 days. The time point of harvest was decided based on microscopy analysis of cell size, cell lysis (50–80%) and YFP fluorescence and varied among different target proteins. For transfection of suspension cells, Sf9 cells were diluted in serum-free Ex-Cell420 to 0.8 × 10^6^ cells/ml 3–4 h prior to transfection. Linear 40 kDa polyethylenimine PEI-MAX (Polysciences, Eppenheim, Germany, Cat No 24765) was prepared in water at 1 mg/ml, pH was adjusted to 7.0 and aliquots were stored at −20 °C [22]. The amount of bacmid DNA used for PEI – DNA complex formation was chosen according to previous experience with transient transfection of insect cells. 1 μg bacmid DNA/ml culture was diluted in 100 μl prewarmed PBS and vortexed gently. PEI used at different DNA:PEI ratios 1:2, 1:4 and 1:6 was pipetted directly into the DNA solution (2 μl, 4 μl, 6 μl/ml culture respectively) and vortexed immediately and vigorously for 3 × 3 s. Complex formation was allowed for 20–30 min at room temperature and the solution was then added dropwise to the cells. The protocols for transfection and PEI preparation were adapted from protcols originally established for HEK293E cells [22]. Transfected cultures at 10 to 100 ml scale were incubated at 26 °C at 120 rpm until virus supernatant P0 was harvested 5 days post-transfection. Baculoviral infection was monitored by recording cell number, viability and diameter as well as YFP fluorescence. For virus amplification, Sf9 cells were diluted in serum-free Ex-Cell420 to 0.5 × 10^6^ cells/ml 3–4 h prior to infection. Sf9 cells at 10 to 100 ml scale were infected with P0 virus in the range of 1:1000 to 1:5000 and incubated at 26 °C at 120 rpm. Virus supernatant P1 was harvested 4 days post-infection. Baculoviral infection was monitored by recording cell number, viability and diameter as well as YFP fluorescence.

### Protein expression

For protein expression, Hi5 and SF9 cells were diluted in serum-free Ex-Cell420 to 1 × 10^6^ cells/ml. As tested in previous experiments the amount of 80–100 μl P0 or P1virus / 10 ml expression culture resulted in maximal YFP fluorescence as well as target protein expression. Virus amounts had to be increased in case expression levels had dropped due to virus decay. For small scale expression tests, 10 ml infected Hi5 and Sf9 cells were incubated at 20 °C and 26 °C at 120 rpm and harvested after 72 h and 96 h. A production scale of 100 ml for constructs #1–4 and 200 ml for constructs #5 und 6 was chosen for this study. Based on small scale expression tests, production of all constructs was performed in Hi5 at 20 °C for 96 h.

### Protein purification and protein detection

All protein sampled were analyzed on 13.5% Amersham Easy SDS-PAGE gels, visualized by Cy5 staining and quantified by the Amersham WB software. For Western Blot analysis, proteins were separated on 4–12% Bis-Tris Nu-PAGE (Life Technologies), blotted on Immobilon-P PVDF (Millipore, Schwalbach, Germany) and visualized with HisProbe™-HRP (Thermo Scientific, Munich, Germany). Protein quantitation was performed by Bradford staining (Pierce, Thermo Scientific, Munich, Germany). For analysis of expression levels in total lysates, 200 μl cells were pelleted and lyzed in 20 mM Tris pH 8.0, 0.25% SDS. Buffers used for protein purification were 50 mM Tris, 500 mM NaCl, 2 mM MgCl_2,_ 10% glycerol, 1 mM TCEP, pH 8.0 with imidazole 10 mM (lysis), 20 mM (wash) and 250 mM (elution). 0.5% Chaps was included in all buffers for purification of #3. Culture supernatants from cells expressing the secreted constructs #5 and #6 were loaded on 0.25 ml cOmplete His-Tag Purification Resin (Roche, Cat No 5893682001), washed in 5 ml wash buffer and eluted in 1 ml elution buffer. Cell pellets from cultures expressing # 1 to #4 were resuspended in 10 ml lysis buffer including AEBSF-HCl (1 mM), Aprotinin (2 μg/ml), Leupeptin (1 μg/ml) and Pepstatin (1 μg/ml), lyzed in a Dounce homogenizer and treated with 750 U Benzonase (Novagen, Cat No 70664–3) on ice for 30 min. Cell extracts were centrifuged for 30 min at 20.000 rpm at 4 °C. Lysate supernatants of #1 and #2 were loaded on 0.25 ml Ni Sepharose High Performance beads (GE, Cat No 17–5268-02), #3 and #4 were loaded on 0.15 ml MagneHis Particles (Promega, Cat No V8560), rotated for 60 min at 4 °C, washed in 5 ml wash buffer and eluted in 0.75 ml (#1 and #2) and 0.45 ml (#3 and #4) elution buffer.
